# Quantitative SPECT imaging and biodistribution point to molecular weight independent tumor uptake for some long-circulating polymer nanocarriers

**DOI:** 10.1039/c7ra09183d

**Published:** 2018-02-01

**Authors:** V. Schmitt, C. Rodríguez-Rodríguez, J. L. Hamilton, R. A. Shenoi, P. Schaffer, V. Sossi, J. N. Kizhakkedathu, K. Saatchi, U. O. Häfeli

**Affiliations:** The University of British Columbia, Faculty of Pharmaceutical Sciences 2405 Wesbrook Mall Vancouver BC V6T1Z3 Canada urs.hafeli@ubc.ca kathy.saatchi@ubc.ca; Department of Physics & Astronomy, The University of British Columbia Vancouver BC Canada; Centre for Comparative Medicine, The University of British Columbia Vancouver BC Canada; Centre for Blood Research, Department of Pathology and Laboratory Medicine, The University of British Columbia Vancouver BC Canada; TRIUMF 4004 Wesbrook Mall Vancouver BC Canada; Department of Chemistry, The University of British Columbia Vancouver BC Canada

## Abstract

Polymeric nanocarriers are promising entities for cancer diagnosis and therapy. The aim of such nanocarriers is to selectively accumulate in cancerous tissue that is difficult to visualize or treat. The passive accumulation of a nanocarrier in a tumor through extravasation is often attributed to the enhanced permeation and retention (EPR) effect and the size and shape of the nanocarrier. However, the tumor microenvironment is very heterogeneous and the intratumoral pressure is usually high, leading to different opinions about how the EPR of nanocarriers through the irregular vasculature of a tumor leads to accumulation. In order to investigate this topic, we studied methods for the determination of pharmacokinetic parameters, biodistribution and the tumor uptake of nanocarriers. More specifically, we used non-invasive quantitative Single-Photon Emission Computed Tomography/Computed Tomography (qSPECT/CT) imaging of hyperbranched polyglycerols (HPGs) to explore the specific biodistribution and tumor uptake of six model nanocarriers in Rag2m mice. We were interested to see if a distinct molecular weight (MW) of nanocarriers (HPG 25, 50, 100, 200, 300, 500 kDa) is favoured by the tumor. To trace the model nanocarriers, HPGs were covalently linked to the strong chelator desferrioxamine (DFO), and radiolabeled with the gamma emitter ^67^Ga (EC = 100%, *E*_γ_ = 185 keV (21.4%), 300 keV (16.6%), half-life = 3.26 d). Without the need for blood collection, but instead using qSPECT/CT imaging inside the heart, the blood circulation half-lives of the ^67^Ga labeled HPGs were determined and increased from 9.9 ± 2.9 to 47.8 ± 7.9 hours with increasing polymer MW. Total tumor accumulation correlated positively with the circulation time of the HPGs. Comparing the tumor-to-blood ratio dynamically revealed how blood and tumor concentrations of the nanocarrier change over time and when equilibrium is reached. The time of equilibrium is size-dependent and increases with molecular weight. Furthermore, the data indicate that for larger MWs, nanocarrier uptake and retention by the tumor is size independent. Further studies are necessary to advance our understanding of the interplay between MW and nanoparticle accumulation in tumors.

## Abstract 2

Polymeric nanocarriers are promising entities for cancer diagnosis and therapy.

## Graphical abstract

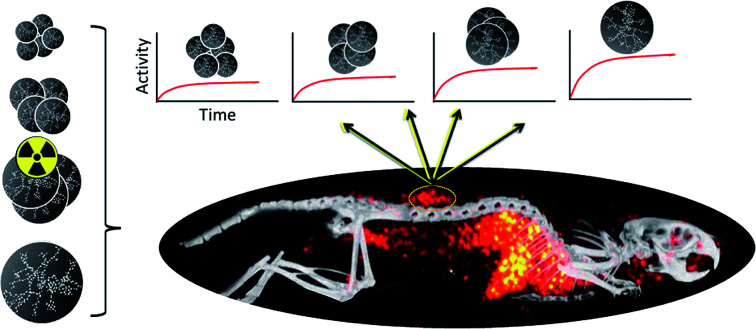

## Introduction

Quantitative SPECT/CT (qSPECT/CT) imaging is a non-invasive technique used to study the distribution and accumulation of radiolabeled nanomedicines *in vivo*. The visualization of radiopharmaceuticals in the body is beneficial for diagnostic and therapeutic applications in cancer research. Due to advances in corrections for photon attenuation and scatter and improved reconstruction algorithms, amongst others, quantitative SPECT data can be produced in units of activity concentration (*e.g.*, kBq mL^−1^). Quantitative SPECT can be as accurate as positron emission tomography (PET), and pre-clinically with even better resolution (0.5 mm *vs.* 2 mm).^1^ Using qSPECT/CT, the distribution and tumor uptake of nanomaterials can be studied in a non-invasive manner with an effort to link nanomaterial properties to circulation characteristics and pharmacokinetics. Factors that are reportedly associated to the success of a nanomedicine are size and shape,^2,3^ because they will affect blood circulation, half-life/clearance and tumor accumulation. Although some dependencies are known, these factors have a difficult and not fully understood relationship.

The reported impact of nanomaterial size on extravasation and retention in tumors has been inconsistent in the literature. Any nanomaterials sized above a hydrodynamic diameter of 5.5 nm are able to escape renal clearance^4^ and sizes between 30 and 200 nm are presumably desired for intratumoral accumulation.^5^ At the lower end of this range, particles can diffuse deep into the tumor, but will not be retained for more than 24 hours, while at the larger end, particles likely reach the extravascular space, but are not able to penetrate further into the tumor.^6^ These explanations might be an oversimplification, and it has been reported that successful nanocarriers likely need to be adjusted for each tumor type, because the tumor microenvironment is heterogeneous and might even vary over time and course of treatment.^7^ At sizes below 20 nm, nanomaterials are often described by their MW in kilo Dalton (kDa). Larger MW nanomaterials reportedly accumulate more in the tumor than smaller MWs,^8^ which is the key motivation for using nanomaterials instead of just small molecule cancer drugs alone.

Accumulation of a nanomaterial in a tumor by the enhanced permeation and retention (EPR) effect is linked to its increased circulation half-life. The longer the material circulates, the more often it passes by the tumor with a chance at each pass to penetrate into the tumor and being accumulated. However, increasing the circulation time can also increase toxicity and accumulation in other tissues. The three major challenges in the design of nanomaterials for therapeutic and diagnostic applications have been named by Choi *et al.*^9^ First, the high background uptake by the mononuclear phagocyte system (MPS) in spleen and liver, second, the lack of complete elimination and associated toxicities, and third, achieving a small hydrodynamic diameter (*e.g.*, 5.5 nm) that allows for a rapid equilibration between intra- and extravascular space.^9^ If the extravascular space is the tumor, several characteristics must be considered. The endothelium of tumor blood vessels can have fenestrations between 100 and 780 nm and allow nanocarriers to extravasate, but tumor heterogeneity, extracellular matrix and increased interstitial pressure can counterbalance the effect of nanocarrier retention.^10,11^ In fact, passive tumor accumulation of nanocarriers by EPR does not often work in humans.^12^ Only a few tumors (Kaposi sarcoma, tumors of head and neck) have shown EPR in the clinic. The heterogeneity of fenestrations in tumor endothelium, the heterogenous pericyte coverage, variable basement membrane characteristics and extracellular matrix have been identified as some of the major problems.^12^ A recent review on nanoparticle delivery into solid tumors reports that the median percentage of administered nanoparticles delivered to the tumor might be as low as 0.7%.^13^ Nonetheless, specific design considerations for tumor accumulation appear frequently in the literature,^7,9,14^ and some already focus on methods to improve or even bypass the EPR effect.^15^ This “dark side” of the EPR and ways to mitigate it have been addressed in a recent article by Huynh *et al.*^15^ Several strategies are proposed for enhanced permeation and delivery including the enzymatic *in situ* formation of nanoparticles in tumors and the use of microbubbles and ultrasound.

The tumor microenvironment is difficult to categorize and the positive aspects of EPR might be overrated or translate poorly into the clinic. Here, we introduce another piece of evidence that relating a specific nanomaterial characteristic (*e.g.*, size or MW) to tumor accumulation might be problematic and depend on the time of measurement. Furthermore, we describe the advantages of quantitative SPECT imaging for nanomaterial distribution studies.

We used the polymer hyperbranched polyglycerol (HPG) as a model nanomaterial tagged with the gamma emitter ^67^Ga and studied size-dependent circulation half-life, biodistribution and time-dependent tumor accumulation. We selected this model nanocarrier, because HPGs are stable macromolecules that can be easily synthesized in a one pot reaction and have a narrow size distribution.^16–18^ HPGs have been used in a number of biomedical and tissue engineering applications including drug delivery and scaffolding applications.^19–23^ They are biocompatible, highly water soluble macromolecules,^24–26^ and several biodegradable versions of HPG have recently been prepared.^27–30^ HPG's three dimensional structure offers many possibilities to modulate their physicochemical and biological properties. A number of modifications are possible on their terminal hydroxyl groups and an introduction of hybrid modifications using nanoparticles, carbon nanotubes or quantum dots were recently reported.^31^ In addition, these polybranched structures have been used for SPECT imaging after binding ^67^Ga or ^111^In, and Gd(iii) for magnetic resonance imaging (MRI).^32–34^ In this study the HPGs were modified with a strong chelator for ^67^Ga called desferrioxamine (DFO) to be visualized and quantified using SPECT/CT.

## Experimental

### Materials

Chemicals and reagents were purchased from Sigma-Aldrich Canada Ltd. (Oakville, ON) and used without further purification unless specified. Glycidol (96%) was distilled under reduced pressure and stored at 4 °C before use. Trimethylolpropane (TMP) was obtained from Fluka (ON, Canada). For animal procedures, isoflurane (AERRANE®) from Baxter Corp., Mississauga, ON, CA was used. Amicon® microconcentrators of various MW cut-off (10, 30, 50 and 100 kDa) and phosphate buffered saline (Gibco™ PBS 7.4) were purchased from Thermo Fisher, CA. ITLC TEC-CONTROL strips (#150-771, dark green) were from Biodex, Shirley, NY, USA.

### Synthesis and characterization of the model SPECT/CT nanomaterials

#### Synthesis of DFO–HPG

The six model nanocarriers are hyperbranched polyglycerols (HPGs) conjugated with the Gallium 67 (^67^Ga) chelator desferrioxamine (DFO) and will be referred to as ^67^Ga–DFO–HPGs followed by their molecular weight (MW) in kDa.

HPGs were synthesized using Schiff-base chemistry as previously described.^35^ HPGs of different MWs (25, 50, 100, 200, 300 and 500 kDa) were created by an anionic ring-opening multi-branching polymerization of glycidol by a single step synthesis.^17,36,37^ DFO was covalently conjugated to the HPGs using a previously published method.^35^

#### Characterization of DFO–HPG

Absorbance spectra were recorded on a Varian (Cary 400 series) UV-vis spectrophotometer and the nanocarriers characterized as to the number of DFO molecules per HPG using the same instrument.^35^ The absolute MW of the DFO–HPGs and hydrodynamic size were determined by gel permeation chromatography (GPC) using a DAW HELEOS II multi angle laser light scattering (MALLS) detector (Wyatt Technology Inc.), an Optilab T-rEX refractive index detector and a quasi-elastic light scattering (QELS) detector (Wyatt Technology Inc. CA) in 1.0 N NaNO_3_ (pH 8) aqueous solution by following the protocols described previously.^35^

#### Radiolabeling of DFO–HPG

All DFO–HPGs were radiolabeled as follows: ^67^GaCl_3_ (185 MBq, 5–10 μL in 0.1 N HCl) was added to DFO–HPG (2.5 mg) in an NH_4_OAc solution (100 μL, 0.1 M). The reaction mixture was stirred at room temperature for 1 h (600 rpm).

^67^Ga–DFO–HPGs were purified using centrifugal filters (Amicon® Ultra 0.5 mL) of the relevant MWCO for each size of DFO–HPG (10 kDa for HPG-25, 30 kDa for HPG-50 and HPG-100, and 100 kDa for HPG-200, HPG-300 and HPG-500). The filtered product was washed twice with 500 μL of H_2_O and PBS 7.4 each and resulted in 50–80% of the activity being collected from the microconcentrator. The collected ^67^Ga–DFO–HPGs were diluted to 600 μL with PBS 7.4 for animal injection (150 μL per mouse). For the sake of comparison, the resultant ^67^Ga–DFO–HPGs contained a similar number of DFOs per milligram of polymer.

#### Labeling efficiency of ^67^Ga–DFO–HPGs

Radiochemical purity and labeling efficiency was measured with instant thin layer chromatography (ITLC) using PBS or saline as the mobile phase. The ^67^Ga–DFO–HPG complexes which have larger molecular weight, would remain at the origin while the free ^67^Ga^3+^ moves with the mobile phase at the solvent front (R_f_(^67^Ga–DFO–HPG) = 0, R_f_(free ^67^Ga^3+^) = 1).

#### *In vitro* stability of ^67^Ga–DFO–HPGs

The stability of ^67^Ga–DFO–HPG in PBS was determined at different time intervals using ITLC in the presence of an excess of EDTA (0.1 M). Briefly, 5 μL of the radiolabeled pure solution (^67^Ga–DFO–HPG) was added to 1 mL of EDTA solution (pH 7.4) and mixed at 37 °C and 650 rpm on an Eppendorf thermal shaker. The resultant solutions were incubated for 1 h and 24 h and analysed by ITLC using a phosphor imager (Cyclone, Canberra Packard, Mississauga, CA). To measure EDTA transchelation the radioactive intensities on the ITLC were integrated and compared (R_f_ (^67^Ga–DFO–HPG) = 0, R_f_ (^67^Ga–EDTA) = 1).

### Long-term *in vitro* stability of ^67^Ga–DFO–HPGs in mouse plasma

The stability of ^67^Ga–DFO–HPG in mouse plasma was determined over 8 days using ITLC. For this purpose 50 μL of ^67^Ga–DFO–HPG tracer in PBS was incubated with 250 μL of mouse plasma at 37 °C (Eppendorf thermal shaker, 650 rpm). Aliquots were taken at designated time points (1 h, 1 d, 4 d and 8 d) and measured by ITLC using 0.1 M EDTA (pH 7.4) as eluent. Radioactive intensities on the ITLC were integrated and compared (R_f_ (^67^Ga–DFO–HPG) = 0, R_f_ (^67^Ga–EDTA) = 1).

### Mouse model

The current study was performed in accordance with the Animal Care Committee of the University of British Columbia under the approved protocol A12-0172. Six groups of 4 immunodeficient female Rag2m mice (homozygous mutation leading to a deficiency of mature B or T lymphocytes)^38^ bearing HER-2 (+) JIMT-1 tumors of 3 to 5 mm diameter^39^ on their back were obtained from the BC Cancer Research Centre. Mice (aged 6 to 10 weeks) had been inoculated subcutaneously with 4 × 10^6^ tumour cells suspended in Matrigel® (1 : 1) above the flank unilaterally and tumours grew for 4 weeks before mice were used for the experiment. Mice were anesthetized using isoflurane on a precision vaporizer (5% in oxygen for induction, between 1.5 and 2.5% in oxygen for maintenance) and received a subcutaneous injection of lactated Ringer's solution (0.5 mL) for hydration prior to each imaging scan. After the induction of anesthesia, an injection of 150 μL of ^67^Ga–DFO–HPG in PBS was administered *via* the tail vein. The average injected activity was 565 μCi. Immediately after injection, the animal was prepared for SPECT/CT imaging on a preclinical small animal scanner. Respiratory rate and temperature were monitored constantly during the scans, and isoflurane and bed temperature adjusted accordingly. Animals were recovered after each scan and sacrificed following the final scan 8 days post-injection. Blood was collected by cardiac puncture and a full biodistribution performed.

### SPECT/CT parameters and image reconstruction

Dynamic whole-body images were acquired during 40 min using a multimodal SPECT/CT scanner (VECTor/CT, MILabs, The Netherlands) equipped with a UHR-RM 1 mm pinhole collimator.^40^ 20 frames of 2 min were acquired for the 40 min scan. Thereafter, acquisitions were done at 24, 96 and 192 h post-radiotracer injection using a single frame of 40 min. Following each SPECT acquisition, a whole-body CT scan was acquired to obtain anatomical information and both images were registered. The ^67^Ga photopeak window was centered at 96 keV with a 20% energy window width and two 10% wide scatter windows were applied on each side to implement scatter correction methods.^41^ For quantitative analysis, SPECT data were reconstructed with pixel ordered subsets expectations maximization logarithm (P-OSEM)^42^ using 10 iterations of 16 subsets and an isotropic 0.4 mm voxel grid. The images were decay corrected to the start scan time and after CT registration, attenuation correction was applied. For visual representation, the reconstructed volumes of SPECT scans were post-filtered with a 3D Gaussian filter. In order to relate the scanner units (counts/voxel) to radioactivity concentration, a calibration factor was determined scanning a source with a known concentration of ^67^Ga.

### Quantitative volume of interest (VOI) analysis and calculation of standardized uptake values (SUVs)

Volumes of interest (VOIs) were manually defined (π.mod, VIEW, Version 3.6) to determine the time activity pattern per target organ. Thus, the delineated regions were inside the heart, liver, lung, kidney, bladder and tumor. The average organ activity per volume was obtained from the SPECT images and the Standardized Uptake Value (SUVs) was extracted from each organ using the following formula.
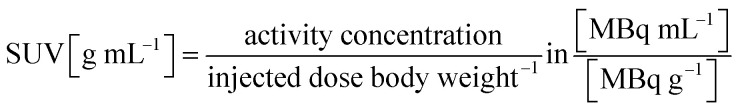


The whole body VOI to measure the total body activity was an oblong sphere enclosing the whole mouse body. The background activity was measured using a 0.47 mL VOI outside the mouse body.

### Biodistribution

A full biodistribution was routinely conducted (blood, heart muscle, liver, kidneys, lungs, small intestine, brain, bladder, muscle, spleen, stomach, bone, tumor, and pancreas) after the last scan on day 8 (192 h). Organs were cleaned from blood, weighed and the activity determined using a γ-counter (Packard Cobra II auto-gamma counter, Perkin Elmer, Waltham, MA, USA). The calibration factor for 37 kBq of ^67^Ga was 1 524 228 cpm (instrument specific). High activity organs (blood, liver) were measured in an Atomlab 500 dose calibrator (Biodex, Shirley, NY, US) if the activity exceeded 37 kBq. Total organ weights were used for the calculations of injected dose per organ (% ID per g organ) except for blood, liver, muscle, bone and pancreas, where average literature values were used.^43–45^

### Pharmacokinetic analysis

To determine the half-lives of the different ^67^Ga–DFO–HPGs, the activities inside the heart and the whole body activities quantified from the SPECT images were used. For the 100, 200, 300, and 500 kDa nanocarriers all 16 data points (*n* = 4, 4 time-point samples) were used. For the 25 and 50 kDa nanocarriers only the first three time-points (*n* = 4, 3 time-point samples) were used. The last time-point (day 8) was excluded because the activity was very close (<2 times) to the measured background activity. The data was fitted to a one or two-compartmental model using the population PK model object of the pharmacokinetic software Phoenix WinNonlin 6.3 (Certara, Princeton, NJ, USA). Initial estimates of the primary PK parameters volume of distribution and rate constants were generated using the optical interface of the software. This interface displays the plots of the dependent variable (activity concentration) *vs.* time according to the parameters of the model. The naive-pooled data approach (NPD) was used to estimate primary PK parameters for the six ^67^Ga–DFO–HPGs. Mahmood *et al.* have shown that even sparse data sets (*e.g.*, *n* = 5, 1 time-point sample) can result in reasonably accurate population estimates of PK parameters using the NPD approach.^46^ Standard errors and coefficients of variation (% CV) were estimated by the software based on the Hessian method of parameter uncertainty. The half-lives were calculated from the primary PK parameter *k* (terminal elimination rate constant) using *t*_1/2_ = ln(2)/*k*.

### Statistical analysis

Data is expressed as mean and standard deviation unless stated otherwise. Significance of the experimental data was assessed using a one-way analysis of variance (ANOVA). The significance level was set to 0.05.

## Results and discussion

### Synthesis and characterization of the model SPECT/CT nanomaterials

The model SPECT/CT nanomaterials of different MWs and varying number of DFO were synthesized as per our earlier report^35^ and characterized using techniques summarized in Table 1. For simplicity the MWs are approximated in this paper and used as 25, 50, 100, 200, 300, 500 kDa. Furthermore, we designed the polymers such that there is on average the same number of chelating DFO groups per mg of polymer (last column of Table 1).

**Table 1:** Characteristics of the DFO–HPG nanocarriers of different MWs^a^

Nanomaterial	MW (Da)^b^	PDI (*M*_w_/*M*_n_)^b^	*R*_h_ (nm)^c^	No. of DFO/polymer^d^
DFO–HPG-25	32 400	1.14	3.3	7
DFO–HPG-50	55 400	1.12	4.1	15
DFO–HPG-100	131 000	1.03	5.4	34
DFO–HPG-200	220 000	1.17	6.4	40
DFO–HPG-300	316 000	1.13	7.3	69
DFO–HPG-500	510 000	1.05	7.8	160

a MW: molecular weight, PDI: polydispersity index, *M*_w_/*M*_n_: weight average MW/number average MW, *R*_h_: hydrodynamic radius.

b Determined by size exclusion chromatography–multi angle light scattering (SEC–MALS).

c Determined by quasi-elastic light scattering (QELS).

d Calculated from UV-vis spectroscopic data.

### Labeling efficiency and *in vitro* stability of ^67^Ga–DFO–HPGs

The resultant DFO–HPG nanomaterials varied in properties depending on their MW and DFO motifs within the polymer scaffold. The nanocarriers with higher MWs (100–500 kDa) showed greater resistance to transchelation when challenged with either transferrin or EDTA compared to those with lower MWs (25 and 50 kDa) as seen in Table 2.

**Table 2:** Radiolabeling efficiency and stability (EDTA) of the ^67^Ga–radiotracers

MW	Labeling efficiency (%)^a^	Transchelation stability 1 h (%)^b^	Transchelation stability 24 h (%)
25	56.2 ± 13.5	60.4 ± 9.9	29.7^c^
50	67.1 ± 10.6	61.5 ± 4.8	39.1 ± 5.8
100	96.4 ± 0.1	92.3 ± 0.7	97.4 ± 0.2
200	97.0 ± 0.4	92.1 ± 1.0	97.6 ± 0.3
300	92.7 ± 3.3	92.4^c^	95.5^c^
500	95.4 ± 5.2	99.5 ± 0.01	99.8 ± 0.1

a The labeling efficiency in % describes the percent bound activity from the initial activity added to the reaction.

b The stability in % describes the ratio of intact ^67^Ga–DFO–HPG to transchelated DFO–HPG.

c *n* = 1, all other measurements *n* = 3.

While the hexadentate DFO generally binds Ga^3+^ with its three hydroxamate groups to form a stable six-coordinate complex (log *β*_Ga–DFO_ = 27.6),^47,48^ our results suggest that the DFOs in the low MW HPGs are positioned in a way, which makes them less strong chelators for ^67^Ga as compared to the larger MW HPGs. The lower molecular weight ^67^Ga–DFO–HPGs were more susceptible to transchelation. While the amount of DFO per mg HPG was kept constant, the total amount of DFOs per molecule was smaller.

### Long-term *in vitro* stability of ^67^Ga–DFO–HPGs in mouse plasma

^67^Ga–DFO–HPGs were stored in mouse plasma over 8 days. The presence of free gallium was very low over 8 days and the corresponding stabilities are given in % in Table 3.

**Table 3:** Stability of ^67^Ga–DFO–HPGs in mouse plasma over 8 days (*n* = 2)

MW	Stability 1 h (%)^a^	Stability 1 d (%)	Stability 4 d (%)	Stability 8 d (%)
50	85.0, 89.7	82.8, 83.5	83.7, 85.9	89.9, 87.1
100	89.8, 91.2	89.8, 87.8	90.1, 85.5	93.4, 92.4
200	90.5, 91.2	86.5, 82.7	90.9, 91.8	95.8, 93.6
300	87.4, 90.2	88.6, 91.6	95.7, 94.3	92.7, 93.0
500	92.0, 82.6	85.3, 82.6	97.6, 94.7	95.8, 84.4

a The stability in % describes the ratio of intact ^67^Ga–DFO–HPG to DFO–HPG.

### Quantitative volume of interest (VOI) analysis and calculation of standardized uptake values (SUVs)

**Fig. 1:**
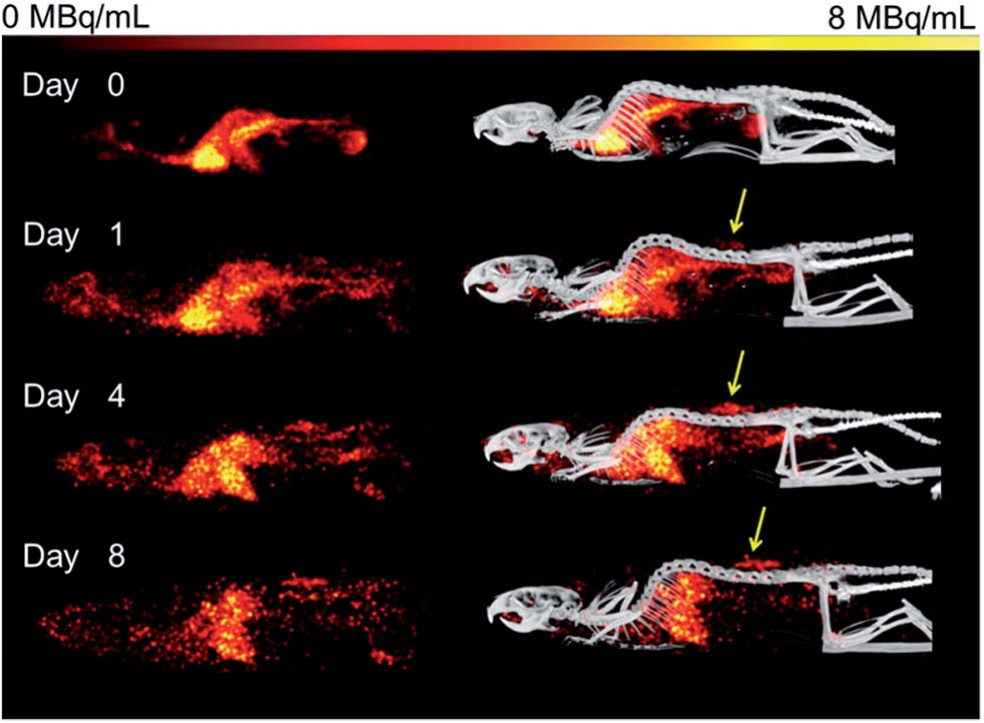
Representative SPECT images of in Rag2m mice bearing subcutaneous (SQ) HER-2 (+) JIMT-1 tumors injected with ^67^Ga–DFO–HPG-200 kDa imaged over 8 days. Left: SPECT images of ^67^Ga–DFO–HPG 200 kDa over 8 days, right: SPECT/CT overlay. Tumor on back is clearly visible (yellow arrow).

Organ activities were quantified from the acquired SPECT/CT images (Fig. 1). The images were clear and the identification of relevant organs was achieved using the co-registered CT image as a guide.

**Fig. 2:**
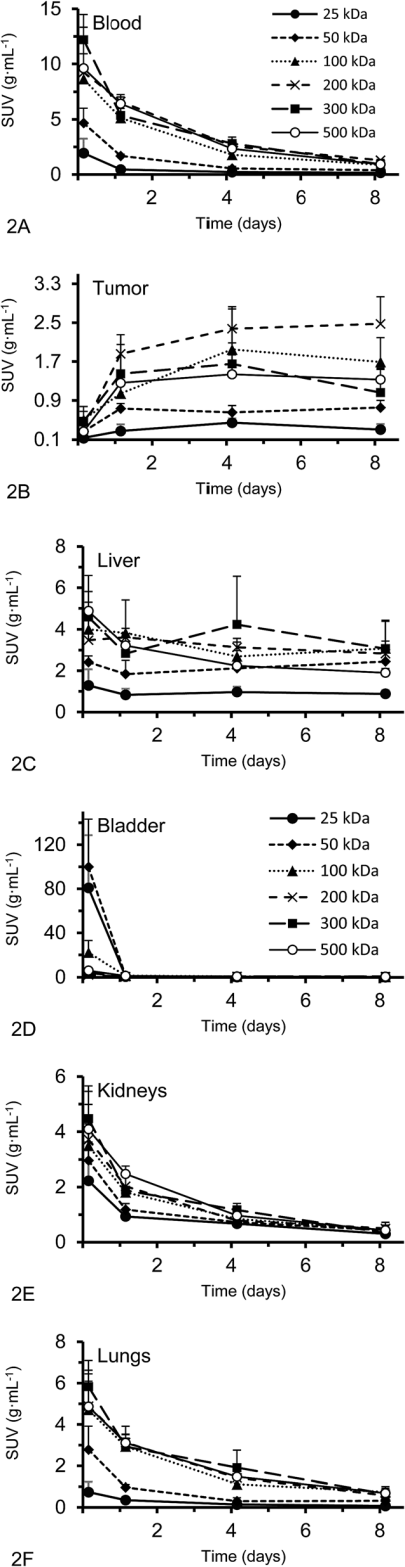
(A–F) Organ and tumor SUVs in g mL^−1^ (average ± SD) of the DFO–HPG nanocarriers with different MW over 8 days; calculated from static SPECT images (*n* = 4). For the tumor, the graph shows a clear accumulation of HPGs, whereas other displayed organs show typical exponential elimination profiles.

Activity concentration data was expressed as standardized uptake values (SUVs) for inside the heart (blood), tumor, liver, lungs, kidney and bladder (Fig. 2). The tumor time-activity data confirms that all DFO–HPG nanocarriers show accumulation in the tumor reaching a maximal concentration at day 4. The well perfused organs heart (blood), liver, lungs and kidneys showed initially high activities but decreased rapidly thereafter. This corroborates the characteristics of a blood pool imaging agent and non-targeted passive organ delivery. Activity differences are most pronounced between the two smaller HPGs (25, 50 kDa) and the three large HPGs (200, 300, 500 kDa), whereas the 100 kDa HPG shows mixed characteristics. The activity curves for the higher MWs (200, 300, 500 kDa) are very close in maximum activity concentration detected and curve shape. The smaller MWs (25, 50, 100 kDa) show an overall lower concentration and uptake but similar curve progressions, except for the bladder. As expected, the 25 and 50 kDa HPGs show rapid and high bladder uptake within the first 40 min (∼80 and 100 g mL^−1^). The 100 kDa HPG also shows high activity in the bladder initially (∼20 g mL^−1^), but otherwise behaves very similar to the three larger MW HPGs (200, 300, 500 kDa) in the quantified organs.

**Fig. 3:**
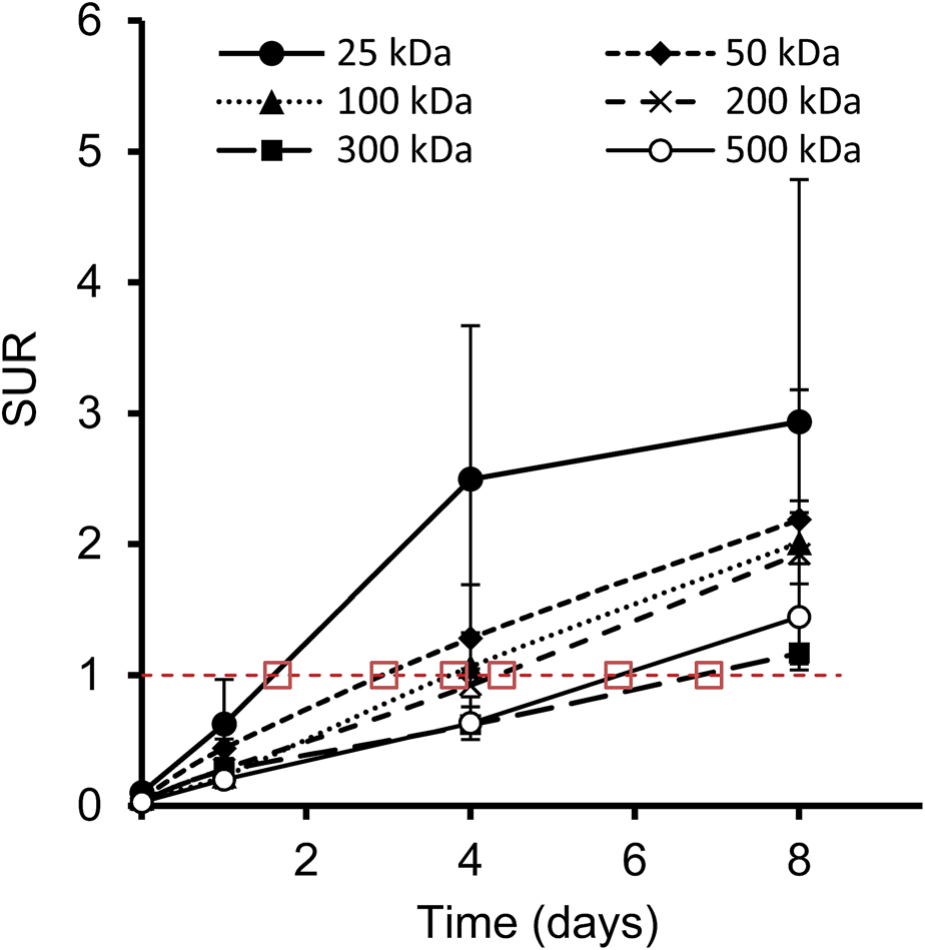
Tumor-to-blood standard uptake ratio (SUR), which is the ratio of the SUVs for tumor and blood, over time; indicated in red is a ratio of 1, where the tumor and blood SUVs are in equilibrium. The marked time points of equilibrium are estimates.

**Fig. 4:**
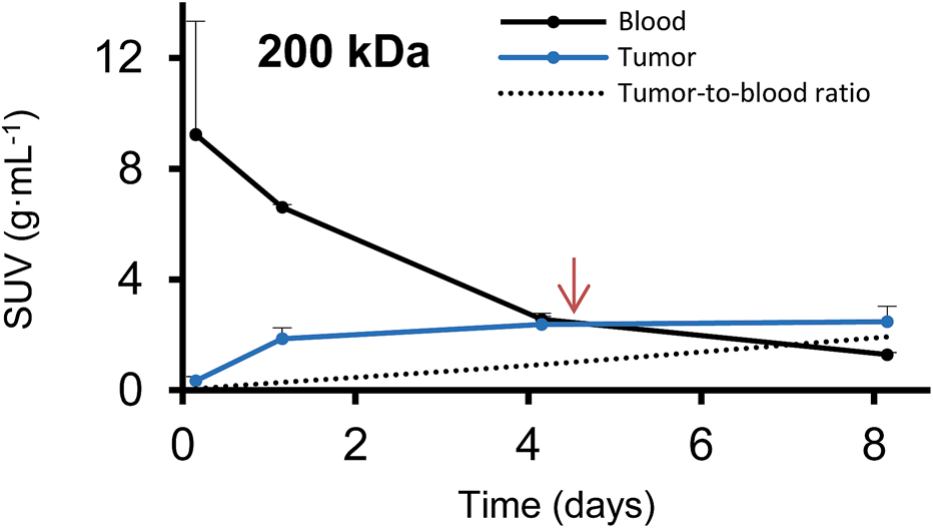
The blood activities (g mL^−1^) and corresponding tumor activities (g mL^−1^) are shown over time for the 200 kDa DFO–HPG. A red arrow indicates the time when the tumor-to-blood ratio reaches 1 at around 4.3 days.

Furthermore, we have determined the tumor-to-blood standardized uptake ratio (SUR)^49^ through a dynamic analysis over 8 days. This value can give an indication of the partitioning of the different HPGs between tumor and blood over time (Fig. 3). It is of particular interest to compare the time it takes to reach a ratio of 1 for each group. The time points are marked in red in Fig. 3 and it is apparent that the time to equilibrium increases with increasing molecular weight from about 1.5 days for the 25 kDa HPG to 7 days for the 500 kDa HPG. In fact, a plot of the equilibrium time from Fig. 3*vs.* the MW indicates a linear relationship with an *R*-squared (*R*^2^) statistic of 0.90. A change in SUR curve progression or slope can either result from a change in blood concentration or tumor concentration. We would like to demonstrate this in detail in Fig. 4, using the 200 kDa DFO–HPG as an example. The disappearance of HPG from the blood and its accumulation in the tumor is shown, and it is apparent that the time to equilibrium is around 4.3 days.

While the SPECT results show dynamic tumor uptake over multiple days (Fig. 2B), the biodistribution data informs about the endpoint of the study on day 8. This single endpoint biodistribution data serves a quite different function than the dynamic SPECT data. It can inform in which other organs a radiolabeled drug accumulates, especially if it is at very low activities that are not apparent by SPECT. This can be very important for the investigation of long-term effects, side effects and toxicities.

Activities measured by SPECT analysis are overall similar to the biodistribution results (see next paragraph). With both methods, the higher MW HPGs showed higher overall tumor uptake. The highest tumor activity with the clearest inter-MW distinction was observed on day 4 and then remained almost constant until day 8. There is no difference between tumor accumulation on day 4 for the 100, 200, 300 and 500 kDa MW and there is no difference between tumor accumulation on day 8 for the 100, 300 and 500 kDa MW (single factor ANOVA, *α* = 0.05).

### Biodistribution

**Fig. 5:**
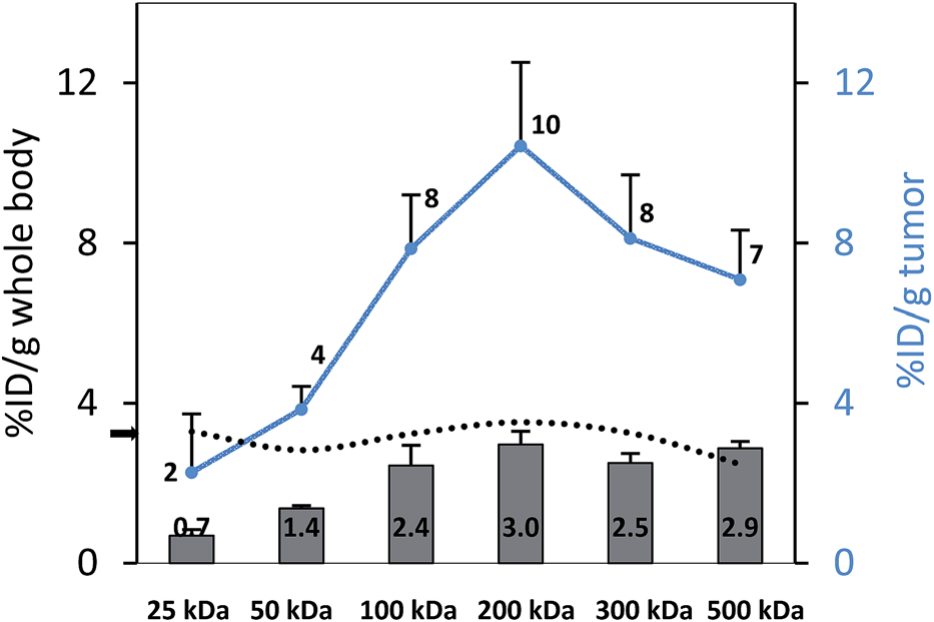
Whole body activities (left axis, % ID per g) and tumor activities (right axis, % ID per g) on day 8 after injection; the difference between tumor accumulation for the 100, 200, 300 and 500 kDa groups is not significant (single factor ANOVA, *α* = 0.05). The tumor-to-body ratio is constant at ∼3 (dotted line, black arrow).

To perform an evaluation of the residence time of the six nanocarriers in the mouse body, the whole body activity was determined on day 8 and compared to the injected dose. The remaining activity (% ID) on day 8 was 17.0, 33.1, 59.3, 72.9, 66.0, 65.3% ID for 25, 50, 100, 200, 300 and 500 kDa groups, respectively. To compare this remaining activity with the tumor activities the numbers were normalized by the body weight to produce % ID per g (Fig. 5, primary axis). The tumor activities (% ID per g) showed the same trend with 2.3, 3.8, 7.9, 10.4, 8.1 and 7.1% ID per g (Fig. 5, secondary axis) as the body activities (% ID per g). Tumor accumulation between the 100, 200, 300 and 500 kDa groups was not significantly different (single factor ANOVA, *α* = 0.05). The tumor concentrations (Fig. 5, blue curve) are above the body concentrations, which points to tumor accumulation. Furthermore, the tumor-to-body ratio (dotted line) is constant at ∼3 across the different groups, which points to a molecular-weight independent constant percentage in the tumor.

**Fig. 6:**
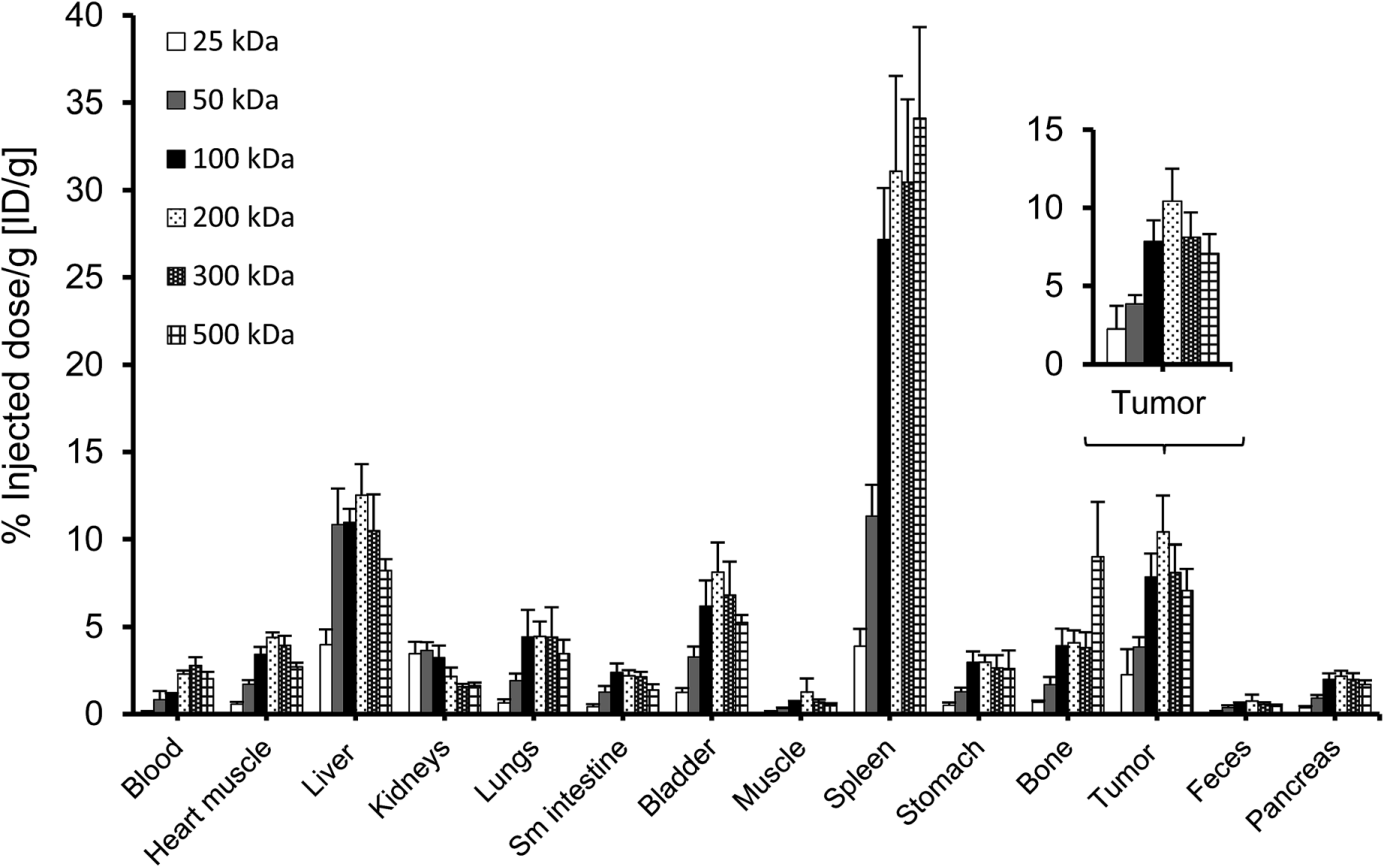
Biodistribution (*n* = 4; average ± SD) of ^67^Ga–DFO–HPG conjugates on day 8 after injection. Enlarged is the tumor distribution: the difference in accumulation across all groups is significant (single factor ANOVA, *α* = 0.05), but not between the larger HPGs (100, 200, 300, 500 kDa).

A complete biodistribution for all relevant organs on day 8 is shown in Fig. 6. Overall, the organs with the highest DFO–HPG concentration were spleen, liver, tumor and bladder. High activities in spleen and liver were expected as pinocytosis may occur here through macrophages and Kupffer cells. We have previously shown that HPGs do not activate complement and have minimal interaction with blood proteins.^32^ Reasons for the increased uptake in spleen and liver can be further investigated. A reason for the very high spleen concentration is that these immunocompromised Rag2m mice have a very small spleen, which at ∼0.015 g is about 10× smaller than normal.

Overall, the study of the distribution of multifunctional nanomaterials can be challenging. Radiolabeled compounds such as our ^67^Ga–DFO–HPG have several possible points of attack that may be determining factors for their resulting organ and tumor distribution. For example, any degradation, cleavage or metabolism of the molecule might result in a distribution that does not reflect the original macromolecule. For this reason, we looked closely at each part of the molecule to determine if the activity detected on day 8 in the various organs likely reflects the distribution of the originally injected nanocarrier. The first part of the molecule is the imaging moiety ^67^Ga. ^67^Ga is chelated by DFO to form a stable six-coordinate complex (log *β*_Ga–DFO_ = 27.6).^47,48^ The stability study of ^67^Ga–DFO–HPGs showed, that ^67^Ga is bound tightly when kept at 37 °C in serum over 8 days. We only found a small amount of bone activity in the biodistribution results for all model nanocarriers which points to some released free Ga^3+^. However, each molecule carries multiple atoms of ^67^Ga and will therefore still be visible in SPECT.

Furthermore, we searched for studies that looked at the metabolism of Fe-DFO or Ga–DFO. Both DFO and Fe-DFO can be enzymatically hydrolyzed over time and transamination or *N*-hydroxylation can take place at the terminal amine.^50^ However, these processes take place over many days, and DFO complexes thus seem to be similarly stable as NOTA (a different Ga chelator) complexes where no *in vivo* degradation has been reported. If there is no metal bound to the DFO, it has been shown that degradation is quite rapid,^48^ however, such un-labeled molecules would be invisible in SPECT and do not have to be accounted for here. The ^67^Ga–DFO part is not the only possible point where degradation can take place in a multifunctional imaging agent. The HPG itself has been reported to be non-biodegradable. There is no hydrolysis or degradation of HPGs *in vitro* in buffer at pH 5 and 7.4 over up to 30 days.^26^

As we cannot know precisely how the tested molecules change after injection, we cannot attribute a certain outcome to an exact polymer size (*e.g.*, “the 500 kDa construct circulates the longest”), but we would rather conclude that constructs that were larger to begin with circulated longer. We are confident that the methods used here can be very valuable to assess distribution in a non-invasive manner and to quickly screen for the distribution and circulation time of particulate systems *in vivo*. Especially for cancer therapy, quantifying the tumor accumulation by *in vivo* imaging, SPECT/CT (or PET/CT, depending on the available radioisotopes) is a valuable tool to determine the time point when tumor activity reaches its maximum, as well as describing the active dose of a drug that reaches the target area.

### Pharmacokinetic parameters

**Fig. 7:**
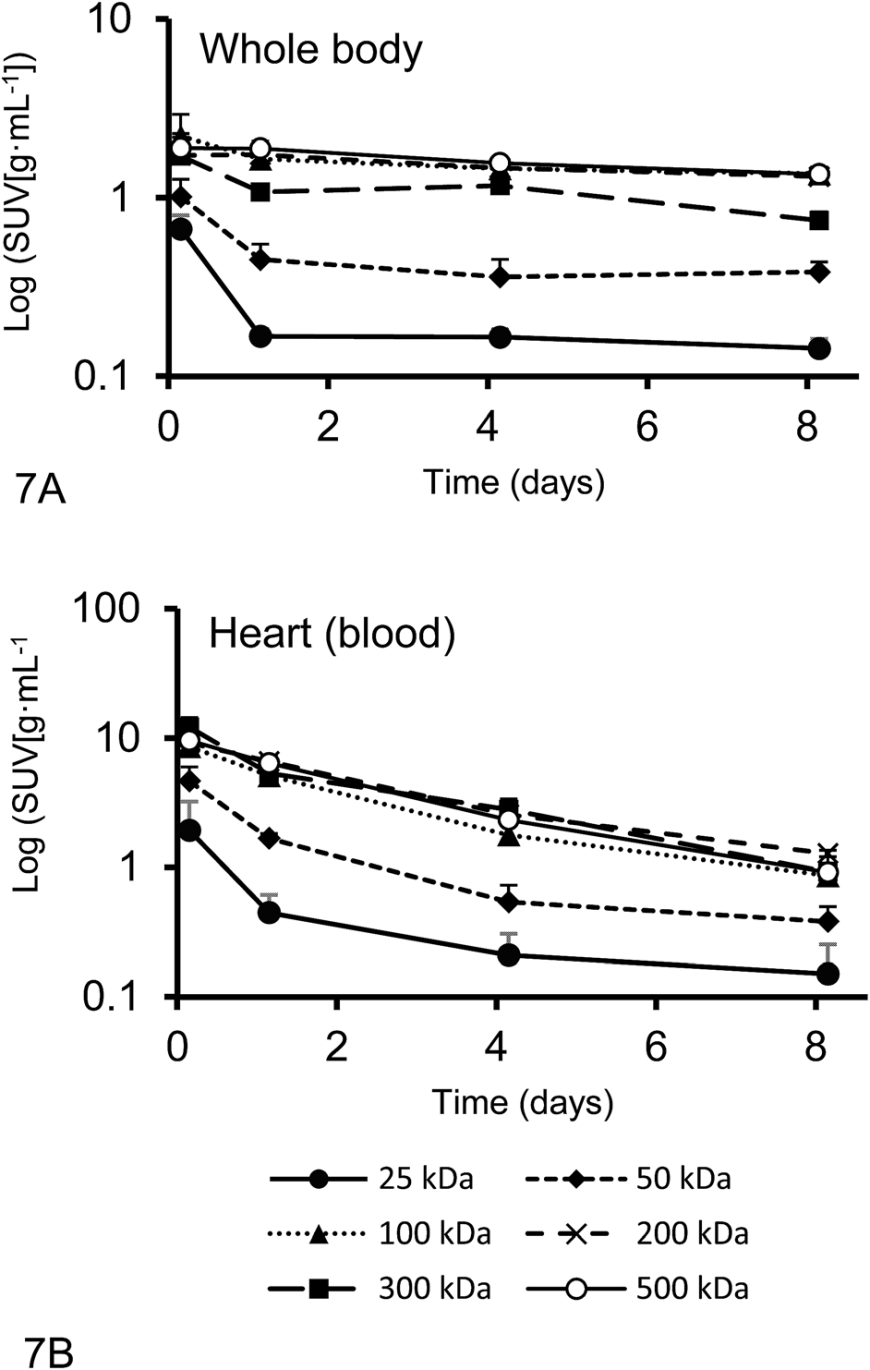
The whole body activities (A) and activities inside the heart (B) (*n* = 4) (average ± SD) from quantitative SPECT data from Rag2m mice bearing subcutaneous (SQ) tumors injected with ^67^Ga–DFO–HPG used for pharmacokinetics (PK) analysis.

The plasma half-live and whole-body half-life of different ^67^Ga–DFO–HPG nanocarriers calculated from inside the heart and whole body activities measured by quantitative SPECT data are shown in Fig. 7. The sampling points were strategically distributed over 8 days to capture the expected residence time of the polymers in the mice.

While the blood circulation half-life of nanomaterials is usually determined using blood sampling, we present an easy method that uses qSPECT inside the heart (*t*_1/2Heart_). By placing the VOIs (*r* = 1.5 mm) inside the heart, the main activity detected is from blood inside the ventricle and excludes the activity of the heart muscle itself. Furthermore we calculated an additional half-life that indicates how long the nanomaterial stays in the body regardless if circulating or not (whole body residence time). We obtained this value using the qSPECT of the whole mouse body (*t*_1/2WB_). These two half-lives for all tested model nanocarriers are summarized in Table 4. As shown here, the heart activity *vs.* time data serves as a sufficient estimator for the macromolecules' blood half-life. In fact, our estimates match the previously reported half-lives for DFO–HPGs closely. A 75 kDa and 637 kDa construct had reported half-lives of 16 and 44 hours,^35^ whereas our 50, 100 and 500 kDa constructs have half-lives of 17, 38 and 48 hours, respectively.

**Table 4:** Half-lives of DFO–HPG nanocarriers for heart and whole body over 8 days

MW	Heart	Whole body
	*t*_1/2Heart_ [hours ± SD]	*t*_1/2WB_ [days ± SD]
25	9.9 ± 2.9	0.5 ± 0.1
50	17.4 ± 4.3	1.9 ± 0.7
100	37.5 ± 10.1	13.0 ± 4.3
200	44.2 ± 5.8	13.6 ± 1.3
300	40.6 ± 14.5	10.4 ± 2.6
500	47.8 ± 7.9	15.2 ± 2.8

### Limitations of the study

This study explores the capabilities and methodology of quantitative SPECT analysis, presents several interpretations of measured SUVs and offers an easy method to extract pharmacokinetic parameters non-invasively. Furthermore, it attempts a comparison with the standard invasive technique for the study of nanomaterial distribution (biodistribution technique). We acknowledge that this study has some limitations. The authors carefully selected nanomaterials of certain size distributions, which are narrow and have PDIs close to 1, but are certainly not perfect. Furthermore, we used one tumor model and did not compare different tumor models for these techniques. However, our methods, findings and interpretations can be easily applied and compared with other tumor models and different nanomaterials in the future, something that is beyond the scope of this manuscript.

## Conclusions

Our study explores the advantages of quantitative SPECT/CT imaging to determine the organ and tumor distribution of radiolabeled nanocarriers. Compared to the classic biodistribution methodology, SPECT/CT does not require sacrificing the experimental animal to dynamically determine organ activities and allows the researcher to determine the amount of circulating radiolabeled nanocarrier at any time. Comparing the tumor-to-blood ratio dynamically can give the researcher an interesting insight into how blood and tumor concentrations change over time and at what time an equilibrium is reached. The time of equilibrium is size-dependent and increases with molecular weight. The VOI analysis employed here is a fast and efficient way to determine pharmacokinetic parameters such as the blood half-life in mice without blood sampling. Monitoring the activity concentration inside the heart and other organs with multiple spherical VOIs placed inside the organs and tumor is an efficient and reproducible way to determine average organ and tumor activities or SUVS.

An interesting trend was observed in the biodistribution data and the SPECT quantifications on day 8. On day 8, a certain amount of nanocarrier is still remaining in the body. If this remaining activity is taken into account, the tumor-to-body ratio is constant at around 3 (Fig. 5), so that one could interpret that the tumor might not take up nanocarriers preferentially based on size. This finding might have no direct therapeutic impact, since for an effective treatment, the total tumor accumulation and not the ratio is of main importance. Larger MW nanocarriers can circulate longer,^51^ and it has also been confirmed in this study that this will lead to higher total nanocarrier amounts in the tumor (*e.g.*, 2.3% ID per g for the 25 kDa *vs.* 10.4% ID per g for the visually best-performing 200 kDa group) (Fig. 5). However, one can hypothesize that complex mixtures of nanomaterials could have an interesting future in an attempt to tailor and maximize tumor accumulation.

## Conflicts of interest

There are no conflicts to declare.

## Supplementary Material
